# Promising Gene Therapeutics for Salivary Gland Radiotoxicity

**DOI:** 10.3934/medsci.2016.4.329

**Published:** 2016-11-30

**Authors:** Renjith Parameswaran Nair, Gulshan Sunavala-Dossabhoy

**Affiliations:** Department of Biochemistry and Molecular Biology, Louisiana State University Health Sciences Center, 1501 Kings Highway, Shreveport, Louisiana 71130, United States of America

**Keywords:** salivary glands, ionizing radiation, aquaporin, TLK1, KGF, VEGF, FGF, PKC delta, HSP, Shh

## Abstract

More than 0.5 million new cases of head and neck cancer are diagnosed worldwide each year, and approximately 75% of them are treated with radiation alone or in combination with other cancer treatments. A majority of patients treated with radiotherapy develop significant oral off-target effects because of the unavoidable irradiation of normal tissues. Salivary glands that lie within treatment fields are often irreparably damaged and a decline in function manifests as dry mouth or xerostomia. Limited ability of the salivary glands to regenerate lost acinar cells makes radiation-induced loss of function a chronic problem that affects the quality of life of the patients well beyond the completion of radiotherapy. The restoration of saliva production after irradiation has been a daunting challenge, and this review provides an overview of promising gene therapeutics that either improve the gland’s ability to survive radiation insult, or alternately, restore fluid flow after radiation. The salient features and shortcomings of each approach are discussed.

## 1. Introduction

Radiation is effective at tumor control, and therefore, radiotherapy remains the mainstay in the treatment of most cancers. However, the unfortunate side-effect of tumor irradiation is the collateral damage to healthy tissues either directly or through bystander effects. Free radicals generated during water radiolysis are the primary agents of damage in cells that lie within the portals of radiation, and biological modifiers released from irradiated cells trigger a response in non-irradiated neighboring and distant cells. A number of genes associated with immune and inflammatory responses in irradiated tissues revealed that NFκB family of transcription factors and their target genes were involved in both normal and tumor tissue responses [1]. More significantly, the induction of immune, inflammatory, and apoptosis genes in non-irradiated tissues residing outside the radiation field indicated that bystander and systemic effects greatly increase the pool of compromised cells well beyond the irradiated region [2].

There are three major salivary glands in humans that are composed of fluid-producing serous or sero-mucinous acinar cells. Saliva secreted by acinar cells passes through the ducts and exits into the mouth where it functions to protect oral tissues as well as facilitate speech, mastication, and swallowing. The inadvertent damage to normal salivary glands during regional radiation invariably results in a reduction in salivary flow that begins within the first weeks of radiotherapy and continues well past the completion of treatment [3]. A study on dose-volume relationship in parotid glands of head and neck irradiated patients found that salivary function is gravely compromised at mean doses >25–30 Gy with no functional recovery within the first year [4,5]. The partial volume thresholds of the parotid gland were determined to be 15 Gy for 67% volume, 30 Gy for 45% volume and 45 Gy for 24% volume [5]. Since standard radiotherapy for head and neck cancer involves exposure to a total dose of 50–70 Gy, a decline in irradiated gland function becomes near certain.

A majority of acinar cells are post-mitotic, and though they are expected to be relatively radio-resilient, they are, in fact, acutely sensitivity to the genotoxin [6,7]. The exact mechanism of salivary hypofunction is not completely clear, but there is general agreement that the initial decline in function is due to the functional incapacitation of the acinar cells, whereas the progressive irreversible loss of fluid output is a result of cell death that is compounded by the inability of the tissue to regenerate [8]. Conventional therapy for salivary dysfunction is inadequate, and a search for gene therapeutics to ward off functional loss began nearly 2 decades ago. The advantages of gene transfer to the salivary glands compared to other organs are obvious. One, salivary glands are exocrine in nature, and they are easily amenable to non-invasive gene transfer via retroductal access [9,10]. Second, localized retroductal delivery directly to the gland minimizes vector dilution and third, it offers an opportunity to target virtually every epithelial cell that lines the ductal tree. Since the first successful demonstration of salivary gland gene transfer, a number of gene therapeutics has been investigated with the hope of offering a better, sustainable solution for treatment of radiation-induced hypofunction [11].

## 2. Approaches to Salivary Gland Gene Transfer

Since naked DNA is inefficient at crossing cell lipid membranes, carriers have been designed to transport genes into cells. Gene carriers can be broadly categorized into viral and non-viral agents. Recombinant viruses are useful tools for gene delivery because of their inherent ability to introduce their DNA into host cells [12]. Genetically altering the viral genome to include the transgene allows its transfer to cells during virus transduction. Replication-deficient recombinant viral vectors that have been used in preclinical salivary gland gene transfer include adenovirus serotype 5, adeno-associated virus (AAV) serotypes 2, 5, and 9, and retroviruses including lentiviruses. Of the 3 common types of viral vectors in salivary gland research (Table 1), adenoviruses are most efficient at transducing dividing and non-dividing cells and establishing rapid and strong gene expression [9]. Immune response to viral proteins however, limits gene expression to a few days and precludes repeat virus administrations [13]. AAV vectors, on the other hand, are less immune reactive and sustained gene expression is realized for a long period [14,15]. AAV is a single-stranded DNA virus, and gene expression is reliant on cellular replication for the generation of a functional double-stranded molecule. As a result, there is a long lag period before transgene expression and a preferential selectivity towards proliferating ductal cells of the salivary glands [15,16]. Wild-type AAV can integrate in the human chromosome 19q13.4, but modern gutless vectors, devoid of all viral genes, greatly lack the ability to combine with the host DNA. As a result, they carry a reduced risk of insertional mutagenesis [17]. Retroviruses are RNA viruses that reverse transcribe their genomes into DNA and then integrate into the host. Retrovirus transduction is, therefore, efficient in mitotically active cells, and stimulation of cell division of latent salivary gland progenitor and stem cells was found to be prerequisite for efficient transduction [18]. As members of the Retroviridae family, lentiviruses too transduce cells by integrating into the host DNA, but integration is favored at actively transcribed sites. This makes lentiviruses uniquely capable of transducing and establishing long-term expression in quiescent cells as well. Human immunodeficiency virus-1 (HIV-1) and feline immunodeficiency virus (FIV) are T-lymphotropic lentiviruses, but unlike the former, the latter displays broad tissue tropism. FIV vectors can transduce most cell types including murine salivary glands to set up lasting gene expression [19]. The lack of pathogenicity in humans and the absence of a cross-reactive immune response in HIV-infected hosts make FIV vectors better suited to clinical applications [20]. Nevertheless, the inherent risk of insertional mutagenesis associated with all retrovirus vectors has, by and large, reserved them for research purposes. The development of non-integrating FIV vectors has increased vector safety, but gene expression from these vectors is transient in rapidly dividing cells [21]. Since most cells of salivary glands are slow dividing or mitotically inactive, it is reasonable to assume that stable salivary gland expression can be realized with non-integrating FIV vectors.

Plasmids are the simplest gene delivery vectors, and their direct transfer carries a low potential for immunogenicity. However, direct transfer of naked DNA to salivary glands has been highly inefficient. Strong salivary gland nucleases rapidly degrade DNA and limit transfection. The use of polyionic aurintricarboxylic acid (ATA), an inhibitor of DNA nucleases, with DNA charge-neutralizing zinc chloride was shown to significantly increase plasmid uptake in rat submandibular glands [23]. However, measurable inflammatory changes to ATA limit its use to basic research. A non-viral agent routinely used to facilitate DNA uptake in cells *in vitro* is cationic lipids. Similar to most cell types grown in culture, cationic lipid-DNA complexes are efficient at transfecting salivary gland cells in serum-free conditions *in vitro*, but are considerably inefficient at DNA transfer to glands *in vivo* [24]. A general reason for the inefficiency is the non-specific adsorption of polyanionic proteins, which restrict interaction of lipid-complexes with cell membranes [25].

Due to the lack of an effectual delivery agent, non-viral transfer of nucleic acids fell out of favor until the demonstration of siRNA and plasmid transfer with microbubble-ultrasound combination [26,27]. Ultrasound causes mechanical perturbation of cell lipid membranes, but when used alongside water-soluble, gas-filled microbubbles, the acoustic pressure waves causes bubble expansion and collapse that transiently disrupts cell membranes allowing the influx of biological molecules. Although microjetting and microstreaming were considered to be major contributors to sonoporation events, some have argued in favor of endosomal entry based on biological uptake of genes in distinct clathrin-coated endocytic vesicles [28]. A number of studies have demonstrated feasibility of microbubble-assisted ultrasound gene transfer in various tissues, and low toxicity and targeted delivery are strengths that make the approach potentially safe for clinical applications [29,30]. Moreover, the availability of equipment and clinical-grade reagents can ease its translation to patient care. Diluting out replication-defective plasmids in slow dividing cells of the salivary glands is less of a concern, and transgene expression after sonoporation has been realized for up to 2 and 4 weeks in porcine and murine salivary glands, respectively [26,31]. Nevertheless, for long-term gene expression, bio-effects of repeated sonoporation need to be assessed, and as of now, ultrasound gene transfer appears well-suited to preemptive salivary gland treatment.

Nanoparticle-based nucleic acid delivery is an attractive approach that has shown promise especially, for siRNA transfer. Nanoparticles are nano-scaled spherical structures made of lipid, polymer, inorganic material or a combination of these that self-assemble with nucleic acids through electrostatic attraction. They can be easily functionalized, but their application has faced challenges with cellular entry and endosomal escape [32]. Advances in nanotechnology, biomaterials, and nucleic acid chemistry have helped overcome the foresaid barriers, and successful siRNA transfer to a variety of cells and tissues including the salivary glands has been reported [33–35]. The use of pH-responsive diblock copolymer nanoparticles that readily bind nucleic acids and promote the destabilization of endosomal membranes increased salivary gland transduction [33]. However, local and systemic toxicities to nanoparticles were observed. Newer polymer-based systems have been shown to be effective carriers of small payloads such as siRNA, but biodegradation, clearance, and toxicity are challenges that need to be tackled before their successful transition to humans.

## 3. Gene Therapeutics for Radiation-Induced Salivary Dysfunction

**Figure 1:**
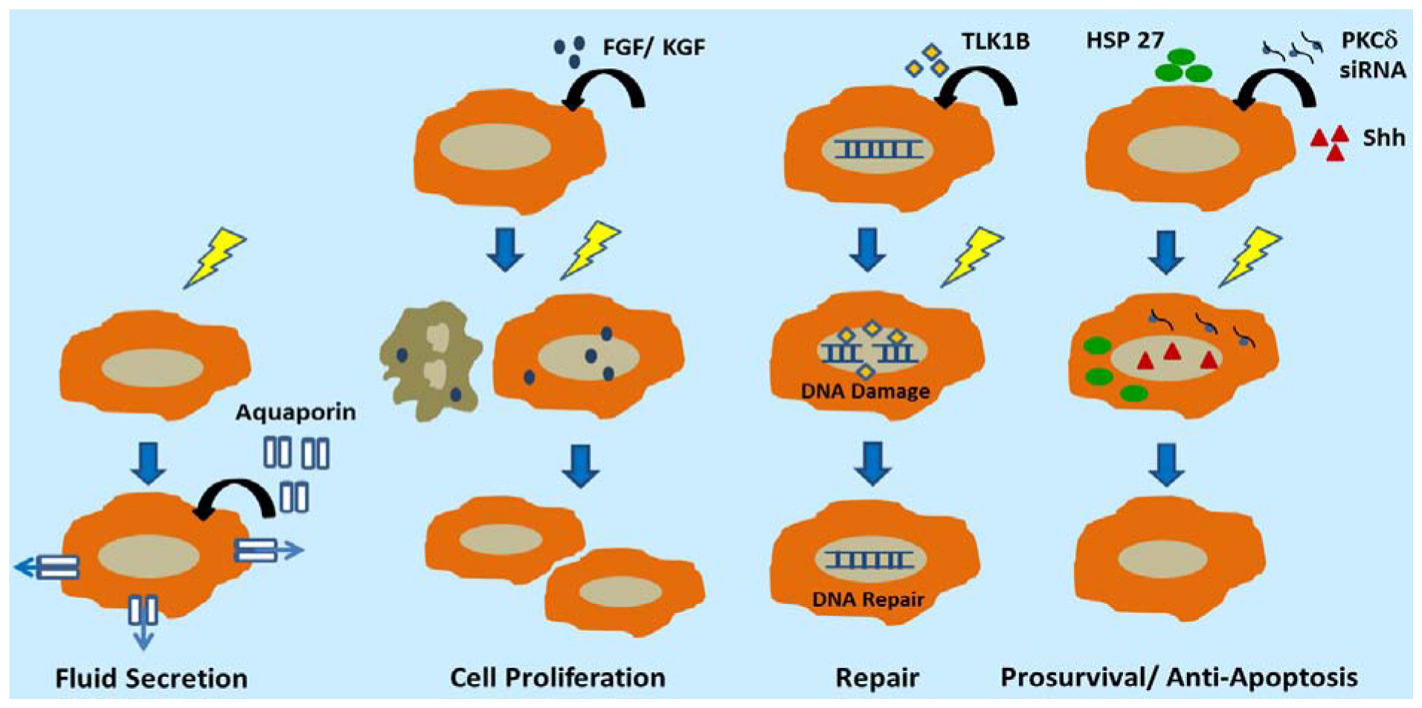
Current gene therapeutics to restore radiation compromised gland function.

Gene therapies that have shown promise in preclinical and clinical studies can broadly be grouped based on their mechanism of action in preventing or reversing salivary hypofunction of radiation (Figure 1).

Based on the mechanism of action, gene therapies are broadly grouped into 4 classes: 1) secretory gene therapy, 2) compensatory growth gene therapy, 3) reparative gene therapy, and 4) prosurvival/ anti-apoptosis gene therapy.

### 3.1. Secretory Gene Therapy

#### 3.1.1. Aquaporin 1 (hAQP1) gene transfer

Aquaporins are a family of membrane-bound proteins that function in the transport of water, solutes and some ions in and out of cells. These water channel proteins are widely distributed in a variety of fluid-transporting epithelial tissues, and they are localized at the luminal and basal membranes in polarized salivary epithelial cells [36]. Aquaporin 1 (AQP1) is predominantly located on endothelial cell membranes, whereas aquaporin 3 and 5 are distributed on the basolateral and apical membranes of human salivary acinar cells, respectively [36,37]. To ameliorate dry mouth, a reasoned approach was, therefore, to increase water permeability of surviving salivary gland cells through aquaporin gene transfer. It was assumed that the osmotic gradient generated by functioning K^+^/ H^+^38 exchangers in surviving salivary ductal cells would move water into the ductal lumen via the aquaporins. Indeed, adenoviral delivery of human AQP1 (Ad-AQP1) in previously irradiated rat submandibular glands was found to increase transcellular fluid flux and restore salivary output to near pre-irradiation levels []. The study was the first successful demonstration of gene therapy in the alleviation of salivary hypofunction. Although a following study on submandibular gland of non-human primates showed mixed results possibly due to the altered distribution of adenoviral receptors on primate salivary gland cells [39], subsequent studies in parotid glands of miniature pigs validated AQP1 expression in improving gland function in a dose-dependent manner [40]. Salivary constituents, however, indicated that K^+^/H^+^ osmotic gradient were not the underlying basis for transcellular fluid movement. Nevertheless, validity of Ad-AQP1 efficacy and confirmation of dose tolerance in rat salivary glands [41] set the stage for its evaluation in patients. In a pioneering single-dose, dose-escalation clinical study (ClinicalTrials.gov Identifier: NCT 00372320) that followed, 6 of 11 patients with radiotherapy-compromised parotid function responded to treatment with measurable reduction in xerostomia [42]. Longer duration of symptom relief after single administration of Ad-AQP1 was unexpected, and a delay in methylation of Ad-AQP1 promoter seen in transduced human salivary gland cells *in vitro* [43] was proposed as the biological mechanism underlying the extended response. Similar to studies in animal [13,44], a localized immune response to the vector was recorded, but otherwise, all patients tolerated virus exposure without life-threatening adverse effects [42]. The results were undoubtedly encouraging, and they compelled the evaluation of AAV as a delivery vector. AAV vectors with a salient advantage of extended gene expression seem better-suited for lasting treatment of previously irradiated glands. After the successful demonstration of AAV transduction of murine and miniature pig salivary glands [14,16] AAV2-AQP1 study in radiation-damaged parotid glands of miniature pig determined that a single virus application effectively reverses hyposecretion up to 8 weeks [45]. An examination of vector toxicity and biospread after virus administration in mouse parotid glands showed that a single virus application was accompanied by minimal gland inflammation, marginal vector spread to neighboring lymph nodes, and development of neutralizing antibodies in blood [46]. Humoral response to the virus raised questions about efficacy of subsequent vector administrations, but stable transgene expression >6 months seen in transduced glands of non-human primates [47] hinted at the possibility of long duration of gene expression in humans. Recruitment for AAV2-AQP1 clinical trial is now underway (ClinicalTrials.gov Identifier: NCT02446249), and we await results of its safety and efficacy in humans.

A recent effort to compare efficacies of Ad-AQP1 and ultrasound-assisted AQP1 gene transfer in pre-irradiated parotid glands of miniature pigs found that restoration of fluid secretion by non-viral transduction was near-similar to adenoviral gene transfer [31]. Salivary output after ultrasound transduction improved up to 2 weeks before declining to levels similar to irradiated controls. Since ultrasound gene transfer is known to evoke a minimalist immune response, the lack of sustained functionality can be a result of non-replicating plasmid dilution in proliferating cells of the ducts and, or, genetic or epigenetic modifications that suppress gene expression *in vivo*. A non-viral approach that elicits a modest inflammatory reaction can potentially permit multiple gene transfers, but safety, feasibility, and cost-effectiveness of life-long treatment necessitates due consideration.

### 3.2. Compensatory Growth Gene Therapy

#### 3.2.1. Basic FGF and VEGF gene transfer

Local blood flow to the salivary glands decreases soon after radiation [48], and the early drop in salivary function that is unaccompanied by epithelial cell loss prompted an inquiry into vasculature damage as the underlying reason for salivary dysfunction. Apart from radiation damage to the fluid producing acinar cells, damage to endothelial cells has been shown to negatively influence salivary function [49]. Basic fibroblast growth factor (FGF) and vascular endothelial growth factor (VEGF) are known mitogens that promote angiogenesis and influence cell growth either directly or indirectly [50,51]. The suitability of FGF or VEGF gene transfer in suppressing radiation-induced functional deterioration of the murine submandibular glands was reported by Cotrim *et al.* [49]. A significant reduction in microvessel density and salivary output caused by radiation was averted by prophylactic adenoviral FGF or VEGF gene transfer. Importantly, extended FGF2 expression from a modified hybrid retro-adenoviral vector was found to better preserve vasculature and offset the effects of radiation on salivary flow in miniature pigs exposed to a fractionated scheme [52]. Growth factors released from transduced salivary epithelial cells can act in a paracrine manner on stromal and endothelial cells, but they can also have an autocrine impact on salivary epithelial cells. Although VEGF is regarded as an endothelial-selective mitogen, evidence of VEGF receptors on salivary ductal cells [53] suggests an additive role of epithelial proliferation in the restoration of salivary function. The potential contribution of growth factor-stimulated epithelial proliferation in allaying hyposalivation should be kept in mind when assessing gland recovery. More importantly, neovascularization is critical to the growth of solid tumors including head and neck carcinomas, and the negative impact of salivary secreted angiogenesis factors on tumor growth and treatment need to be evaluated.

#### 3.2.2. KGF gene therapy

Keratinocyte growth factor (KGF) is an epithelial cell specific growth and differentiation factor that acts through a subset of FGF receptors [54]. Several studies have shown the usefulness of recombinant human KGF in regeneration of damaged salivary gland epithelial cells and its effectiveness in reducing cancer therapy-related mucositis [55,56]. By ingeniously expressing KGF in murine submandibular glands, Zheng and colleagues demonstrated the usefulness of salivary gland-secreted KGF in accelerating repair of radiation-damaged oral mucosa [57]. Moreover, adopting a hybrid adeno-retroviral vector, the group determined that continued KGF expression in salivary glands also protects against fractionated radiation-induced salivary dysfunction [58]. KGF released from transduced cells binds to FGF receptor 2 (FGFR2) on parenchymal, endothelial, and stem/ progenitor cells and stimulates proliferation in an auto-paracrine manner. A major concern with secreted KGF, therefore, is its potential to affect tumor growth. Since no effects on growth or response to radiation of FGFR2-expressing head and neck tumor xenografts were evident, KGF salivary gland gene transfer was suggested to be relatively safe [58]. Palifermin, a truncated human KGF, is the only US Food and Drug Administration (FDA) approved drug for oral mucositis in patients undergoing conditioning therapy before hematopoietic stem cell transplantation. It is anticipated that after conclusive proof of safety of KGF gene transfer to salivary glands, an approval for use in head and neck radiotherapy patients would have dual impact on salivary dysfunction and oral mucositis.

### 3.3. Reparative Gene Therapy

#### 3.3.1. Human TLK1B Gene therapy

Tousled-like kinase 1B (TLK1B) is a normal cellular variant of the full-length TLK1 protein [59]. Translation initiates at a downstream start codon, and as a result, the shorter variant is N-terminal truncated, but otherwise, identical to TLK1. Since protein sequences within the C-terminal kinase domain are identical, the variant, not surprisingly, has been found to target the same substrates namely, anti-silencing factor 1 (ASF1), histone H3, Rad9, and myelin basic protein, and play important roles in DNA replication, chromatin assembly, and DNA damage response and repair [59–66]. The role of TLK1B in radio-protection was first uncovered in mouse breast epithelial cells [59], and kinase activity was found to be essential for the radio-resistant phenotype [65]. The findings of improved radio-resistance in rat salivary gland acinar and ductal cells [67,68] suggested that the role of TLK1B in the process extended to other cell types. Preemptive *in vivo* delivery of adenovirus TLK1B to rat submandibular glands was effective at thwarting functional decline after single-dose irradiation [67], and more significantly, longer duration of TLK1B expression from adeno-associated viral vector serotype 9 (AAV9-TLK1B) guarded against fractionated radiation-induced salivary hypofunction [15]. Unlike adenoviruses that robustly transduce both salivary gland ductal and acinar cells, AAV9, similar to AAV2 and AAV5, was selective at transducing cells of the convoluted granular tubules and the secretory and intercalated ducts [15,16]. The salvation of salivary flow despite the lack of acinar cell transduction suggested that the protection of stem/progenitor cells within ducts is vital to cell replenishment and preservation of function. However, analogous to the risk associated with all radioprotectors, vector spread to cancer cells could undermine their eradication. Measures that provide discriminatory gene expression in normal and cancer cells need to be developed to overcome the shortfall.

### 3.4. Prosurvival/Anti-apoptosis Gene Therapy

#### 3.4.1. PKC delta gene therapy

Most of the gene therapy studies have been focused on exogenous expression of therapeutic proteins in salivary glands that promote cell growth or repair to assuage the effects of radiation. In contrast, suppression of apoptosis through siRNA transfer was recently shown as an alternate strategy in preserving salivary gland function. Protein Kinase C delta (PKCδ) is a ubiquitously expressed cellular variant that when activated controls cell growth, differentiation, and apoptosis [69,70]. The regulation of radiation-induced PKCδ activation through inhibitory phosphorylation was shown to protect salivary glands against radiation [71]. Silencing the pro-apoptotic gene in murine salivary glands through preemptive retroductal administration of PKCδ siRNA-nanoparticle complexes, Arany *et al.* demonstrated significant salvation of salivary gland tissue and function against radiation [33]. Although an immune response to nanoparticles was observed, the use of improved biocompatible and biodegradable nanocarriers can reduce the concern. However, the possibility of inducing genomic instability by by-passing apoptosis in cells with persistent DNA damage needs careful deliberation.

#### 3.4.2. Heat shock protein (HSP) gene therapy

HSPs are evolutionarily conserved molecular chaperones that are named based on their molecular weights. They bind nascent polypeptides to guide protein folding towards stable conformations. They were first identified as being upregulated in response to heat shock, but have, thereafter, been found to be increased also in response to other stresses including ionizing radiation [72,73]. Proteins that are altered or unfolded under stressful stimuli are directly acted upon by HSPs to reestablish proper conformations. Moreover, HSPs have been shown to suppress the activation of caspases and the release of pro-apoptotic factors from the mitochondria to allay the induction caspase-dependent and independent apoptosis. Exogenous expression of individual HSP27 or HSP70 was shown to subvert apoptosis in other systems [74–76], and an investigation in murine salivary glands demonstrated that HSP25, a murine homolog of HSP27, as well as stress-inducible HSP70 gene transfer effectively suppressed radiation-induced cell loss and preserved gland function [77]. Various HSPs work in concert during folding of denatured or naive proteins, and a radioprotective effect of an individual HSP implicates its role in preventing protein aggregation and, or, disabling the induction of cell death pathways. Since apoptosis is a protective mechanism that promotes the clearance of irreparable cells, apoptosis failure can lead to cancer—a risk that needs to be assessed.

#### 3.4.3. Sonic hedgehog (Shh) gene therapy

Shh is a secreted protein that diffuses to form a concentration gradient. It impacts left-right and dorso-ventral neural patterning as well as dorso-ventral axis during vertebrate embryogenesis [78]. Shh acts on cells by binding to and inactivating Patched-1 receptors, which then activates Shh signaling to the downstream Gli zinc-finger transcription factors. Depending on the concentration, time and biochemical nature of the morphogen, different sets of genes are regulated to achieve a myriad of cell types. Apart from the determination of cell fate, Shh also regulates cell growth and proliferation through transcriptional control of cell cycle regulators, and non-canonical signaling through phosphoinositide 3-kinase (PI3K)-AKT prosurvival pathway [79]. In a recent study, adenovirus Shh gene transfer to murine salivary glands was shown to be effective in suppressing radiation-induced gland hypofunction [80]. Salivary tissue and function were preserved despite the transduction of a few cells, which suggest an auto- paracrine influence of secreted Shh and, or, Shh-stimulated growth factors. Uncontrolled activation of Shh signaling has been shown to circumvent establishment of S-phase checkpoint, and increase the risk of genomic aberrations and cancer in Patched-1 haploid insufficiency mice [81]. Although transient Shh expression in salivary glands was found to not induce tumor formation or affect the growth of pre-existing tumors [80], tightly regulable expression vectors would improve safety of Shh gene transfer.

## 4. Conclusion

Stimulating cell proliferation, overriding cell cycle checkpoints, or by-passing apoptosis can heighten the risk of tumorigenesis especially, in context of radiation-damaged stem/progenitor cells of the salivary glands. Additionally, inadvertent transduction of cancer cells with genes that promote growth, suppress apoptosis, or increase repair can affect tumor eradication. Precautions that include gene switches for spatial and temporal regulation of gene expression or mosaic viruses that discriminate between healthy and tumor cells can enhance safety of the therapeutics. Currently, aquaporin gene transfer is the only gene therapeutic that has advanced to clinical trials for radiotherapy-damaged salivary glands. With the demonstration of long-term safety of other approaches, we expect that they too will proceed towards investigation in humans in the near future.

## Figures and Tables

**Table 1:** General characteristics of commonly used recombinant viral vectors in salivary gland gene transfer.

Characteristic	Adenovirus	AAV	Lentivirus
Genome size	36 kb	4.7 kb	9 kb
DNA	ds DNA, linear	ss DNA, linear	ss RNA, linear
Tissue tropism	Broad	Selective	Broad
Infectivity	High	Modest	Modest
Transduction	Dividing and non-dividing cells	Dividing cells	Dividing and non-dividing cells
Packaging capacity	7–8 kb	4.5 kb	8 kb
Transgene integration	No	No	Yes
Transgene expression	Short-lived	Long-term	Long-term
Immune response	High	Low	Low

* adapted in part from Baum *et al.* 2003 [22].

## Abbreviations

AAV: adeno-associated virus
FIV: feline immunodeficiency virus
HIV: human immunodeficiency virus
TLK1: Tousled like kinase 1
KGF: keratinocyte growth factor
FGF: fibroblast growth factor
FGFR2: fibroblast growth factor receptor 2
NFκB: nuclear factor kappa B
VEGF: vascular endothelial growth factor
PKCδ: protein kinase C delta
HSP: heat shock protein
Shh: sonic hedgehog
